# Q fever in the Irish dairy herd

**DOI:** 10.1186/s13620-026-00343-5

**Published:** 2026-04-15

**Authors:** Katie Corridan, Patrick Wall, Guy McGrath, Conor McAloon

**Affiliations:** 1 School of Public Health, Physiotherapy and Sports Science, University College Dublin Belfield, Dublin 4, Ireland; 2 Centre for Veterinary Epidemiology and Risk Analysis, School of Veterinary Medicine, University College Dublin Belfield, Dublin 4, Ireland

**Keywords:** Coxiella burnetii, Q fever, Coxiellosis, herd-level prevalence, Bulk tank milk, ELISA, Zoonosis

## Abstract

**Introduction:**

*Coxiella burnetii*, the causative agent of Q fever, is a notifiable zoonotic pathogen in Ireland. While typically subclinical in ruminants, infection is associated with reproductive losses. In humans, disease can range from asymptomatic to more serious complications. Ruminants have been identified as the main reservoir for human infection. Ireland’s dairy cattle industry has expanded substantially in recent years, yet current data on the national prevalence of *Coxiella burnetii* in dairy herds are limited. Understanding the herd-level prevalence and associated risk factors is essential for informing disease management and control strategies.

**Methods:**

Bulk milk tank testing results from an Irish dairy cooperative herd health programme were analysed to determine the apparent and true prevalence of *Coxiella burnetii* antibodies, using 2022 data. Further analysis was conducted to determine the relationship between *Coxiella burnetii* prevalence and co-morbid disease and herd characteristics.

**Results:**

2687 dairy herds were included in the sample. The true prevalence of *Coxiella burnetii* antibodies was 61.7%. *Coxiella burnetii* prevalence was associated with increasing herd size, whereas higher herd average EBI and lower replacement rates were associated with decreased odds of infection.

**Conclusion:**

This study provides updated data, revealing the highest herd-level prevalence of *Coxiella burnetii* antibodies reported to date in Ireland. The association with larger herd size is particularly relevant with the substantial growth in the national dairy herd over the past decade. These findings reinforce the need for further research into transmission dynamics, impact on production and zoonotic risk.

**Supplementary Information:**

The online version contains supplementary material available at 10.1186/s13620-026-00343-5.

## Background

Coxiellosis or Q fever is a notifiable zoonotic disease caused by the bacterium *Coxiella burnetii*. Ruminants have been identified as the main reservoir for human infection [1]. In animals, coxiellosis can pose a significant risk to herd health and loss of production through decreased fertility, abortions, and anorexia [2]. In humans, Q fever most commonly presents as a self-limiting flu-like illness but may cause pneumonia and hepatitis in rare cases and has been associated with spontaneous abortion in pregnant women [3]. Infection in humans is frequently subclinical, leading to a consensus in both medical and veterinary literature that Q fever is likely underdiagnosed and underreported [4, 5].

In Qfever outbreaks, human transmission occurs through inhalation of aerosol dust particles [6]. Q fever can also be an occupationally acquired disease transmitted to farmers, their families, staff, and veterinarians, through inhalation of contaminated particles, ingestion or direct contact with birth fluids or placenta. The organism is also shed in milk, urine, and faeces. Factors such as the proximity and intensity of exposure to infected animals or their products, personal hygiene practices, and individual susceptibility further influence transmission risk [7].

While human Q fever outbreaks in the Northern Hemisphere are often linked to intensively housed small ruminants, such systems are limited in Ireland. Consequently, this study focuses on *Coxiella burnetii* prevalence in dairy cattle, which dominate the country’s dairy industry, acknowledging that human health risks from cattle may differ from those associated with small ruminants elsewhere [8, 9] Previous studies have indicated that Q fever is endemic in the Irish dairy herd, with a reported herd-level prevalence of 19.5% from 1,489 randomly selected bulk milk tank samples — slightly above the estimated European average of 15.1% [10]. However, this data is now a decade old. Since then, average herd size in Ireland has increased significantly, and changes in herd management, biosecurity practices, and environmental factors may have influenced disease dynamics. Updated prevalence estimates are therefore needed to better assess current animal and public health risks. Bulk tank milk (BTM) sampling combined with enzyme-linked immunosorbent assay (ELISA) has been shown to be a cost-effective method for estimating national herd-level prevalence [10].

The objective of this study was to estimate the current herd-level prevalence of *Coxiella burnetii* in the Irish dairy cattle herd and to identify associated herd-level risk factors using bulk tank milk ELISA results. These findings will support veterinary and public health decision-making, particularly in the context of occupational and environmental exposure.

## Methods

### Programme/dataset description

This study utilised BMT samples collected as part of an Irish dairy cooperative dairy cattle herd health programme. The study sample comprises all participating dairy cattle herds in the programme in 2022. By their nature, dairy cooperatives are regionalised. Herd owners subscribe to the Herd Health Programme annually for a subscription fee. As part of the herd health programme, participating herds are screened for a panel of infectious disease antigens, including *Coxiella burnetii*, infectious bovine rhinotracheitis (IBR), salmonellosis (*Salmonella enterica*), leptospirosis (*Leptospira Hardjo*), *Neospora caninum*, *Ostertagia ostertagi* (stomach worms), and *Fasciola hepatica* (liver fluke). The programme is open to all Irish milk producers, regardless of their co-operative affiliation.

### Sample testing

The samples used for BMT testing are taken concurrently with routine milk constituent payment testing. BMT samples were collected aseptically from each herd and stored at 4 °C until testing, which occurred within 72 h. BMT samples were tested using the ID Screen Q Fever Indirect Multi-species (Innovative Diagnostics, Grabels), an indirect ELISA designed to detect antibodies against *Coxiella burnetii* in milk samples. Testing was performed according to the manufacturer’s protocol.

### Data processing

Test results were dichotomised to positive or negative test status based on their signal-to-positive ratio (S/P ratio) according to the manufacturer’s guidelines. Herd level variables (e.g. herd size, herd average economic breeding index (EBI), percentage of herd at first lactation) were extracted from the Irish Cattle Breeding Federation for the year 2022 [11].

All data were anonymised and linked to unique herd identification numbers before transfer to the researchers.

### Data analysis

A herd was deemed positive if any one of the BTM samples in that calendar year tested positive. Apparent herd-level prevalence was calculated as the proportion of positive herds among all tested herds in that year. Confidence intervals were estimated using the Clopper-Pearson method [12]. Herd-level true prevalence was estimated using the Rogan-Gladen estimator, with confidence intervals estimated using the delta method [13]. For these calculations, test sensitivity and specificity values of 0.86 and 0.99, respectively, were used based on previous evaluation of the ID Screen ELISA Q Fever test by Paul et al. [14], who estimated these parameters using bulk tank milk samples.

The relationship between herd level vaccination and herd size was explored using a Welch two sample t-test.

Statistical analysis was conducted to identify herd-level factors and antigen status variables associated with bulk tank milk positivity for *Coxiella burnetii*. The outcome variable was binary (positive/negative) and therefore analysed using logistic regression models.

Independent variables were herd size, average herd Economic Breeding Index (EBI), percentage of herd at first lactation, exposure to infectious bovine rhinotracheitis (IBR), salmonellosis (*Salmonella enterica*), leptospirosis (*Leptospira Hardjo*), *Neospora caninum*, *Ostertagia ostertagi* (stomach worms), and *Fasciola hepatica* (liver fluke).

Each variable was first trialled in a univariate analyses, and variables with *P* < 0.20 were considered for inclusion in multivariable models to ensure potentially relevant predictors were not prematurely excluded.

Biologically relevant herd-level variables (herd size, EBI, and first lactation proportion) were first trialled in a core model and then retained in all models to control for potential confounding. Separate multivariable logistic regression models were constructed for each antigen to assess their independent association with *Coxiella burnetii* positivity while adjusting for herd-level confounders.

Model fit was evaluated using the Hosmer–Lemeshow goodness-of-fit test.

All statistical analysis was conducted in R studio version 4.3.0. (R Core Team [15]).

Ethics exemption was granted from UCD Taught Masters Research Ethics Committee.

## Results

### Descriptive statistics

The final dataset for analysis consisted of 2649 herds comprising a total of 224,204 individual cows. Descriptive statistics for herd characteristics included in the sample and the national average as reported by the Irish Cattle Breeding Federation can be seen in Table 1. The median herd size for this sample was 117 lactating females. The median herd percentage at first lactation was 20%, with a range of 0% to 53%. The median herd average EBI was €151, with a range of -€57 to €239.

**Table 1:** Descriptive statistics for herd characteristics

	Study Median	Study Range	National Average
Herd Size	117	1-1,175	107
% Herd at first lactation	20%	0–53%	19%
Herd average Economic Breeding Index	€151	-€57 - +€239	€151

None of the herds included in the sample had been vaccinated for Q fever, but there was a high level of vaccination across the population sample for other diseases. 62.5% herds were vaccinated for IBR, 69.7% herds were vaccinated for leptospirosis, and 57.2% herds were vaccinated for Salmonella. The majority of herds also demonstrated a low parasitic burden. 85.3% of samples were negative for liver fluke and 78% of herds returned either a negative or low positive result for stomach worms.

### Prevalence estimates

Of the 2,687 herds tested, 53.5% (tested positive on bulk tank milk. After adjusting for test sensitivity (86%) and specificity (99%) using the Rogan-Gladen estimator, the true herd-level prevalence was estimated at 61.7% % (95% CI: 59.5%–%).

### Vaccination

A total of 86.5% of herds were vaccinated against at least one antigen, while 13.5% were not vaccinated for any of the antigens studied.

Herds vaccinated against at least one antigen had a significantly larger mean herd size compared to non-vaccinated herds (156 vs. 101 animals; *P* < 0.001).

### Multivariable analysis

There was no significant association found between testing positive for *Coxiella burnetii* on BMT sampling and stomach worm burden (*P* = 0.58) or *Neospora C aninum* (*P* = 0.88). As such, these variables were not included in the multivariable analysis.

The results of the core multivariable analysis are included in Table 2. The results of multivariable analysis models for each antigen are included in Table 3.

**Table 2:** Core Model Multivariable Logistic Regression Analysis

Model	Variable	OR	95% CI	*P* value
*Core Model*	Herd size	1.01	(1.006–1.009)	< 0.001
	Herd average Economic Breeding Index	0.995	(0.993–0.997)	< 0.001
	% First lactation	0.245	(0.103–0.572)	0.001

**Table 3:** Antigen Models Multivariable Logistic Regression Analysis

Model	Variable	Category (Reference)	OR	95% CI	*P* value
IBR	IBR negative	Ref = Vaccinated	0.99	0.8–1.23	0.911
	IBR positive	Ref = Vaccinated	0.64	0.40–0.64	< 0.001
Lepto	Lepto negative	Ref = Vaccinated	0.63	0.48–0.81	< 0.001
	Lepto low positive	Ref = Vaccinated	1.38	0.83–2.32	0.22
	Lepto medium positive	Ref = Vaccinated	0.84	0.62–1.14	0.26
	Lepto high positive	Ref = Vaccinated	1.21	0.92–1.60	0.18
Salmonella	Salmonella negative	Ref = Vaccinated	0.75	0.62–0.90	0.002
	Salmonella positive	Ref = Vaccinated	1.25	0.98–1.60	0.069
Liver Fluke	Liver Fluke Positive	Ref = Negative	0.69	0.55–0.86	< 0.001

The core multivariable model identified three significant predictors of *C. burnetii* positivity (Table 2). Increasing herd size was associated with higher odds of seropositivity (OR = 1.01; 95% CI: 1.006–1.009; *P* < 0.001). Higher herd average EBI was associated with reduced odds of seropositivity (OR = 0.995; 95% CI: 0.993–0.997; *P* < 0.001). A greater proportion of first-lactation animals was associated with reduced odds of seropositivity (OR = 0.245; 95% CI: 0.103–0.572; *P* = 0.001).

The *antigen-specific models* are summarized in Table 3. Key findings included herds negative for IBR had lower odds of *C. burnetii* positivity compared with vaccinated herds (OR = 0.64; 95% CI: 0.40–0.64; *P* < 0.001). Herds negative for leptospirosis had lower odds of *C. burnetii* positivity (OR = 0.63; 95% CI: 0.48–0.81; *P* < 0.001). Herds negative for salmonella had lower odds of *C. burnetii* positivity compared with vaccinated herds (OR = 0.75; 95% CI: 0.62–0.90; *P* = 0.002). Herds positive for liver fluke had reduced odds of *C. burnetii* seropositivity (OR = 0.69; 95% CI: 0.55–0.86; *P* < 0.001).

Model fit was evaluated using the Hosmer–Lemeshow goodness-of-fit test. All five multivariable models demonstrated adequate fit (P-values 0.25–0.87), and diagnostic plots showed no violations of model assumptions.

## Discussion

The large sample population of 2,687 dairy herds included in this study represent nearly 15% of the total number of dairy herds in Ireland as per the Irish Cattle Breeding Federation. The sample population was relatively well matched with the national average in terms of, herd size, replacement rate and genetic merit [11].

In this study the apparent prevalence for *Coxiella burnetii* was 53.5% rising to 61.7%% after adjusting for ELISA sensitivity and specificity [14]. Earlier Irish bulk-tank ELISA studies reported a true prevalence of 19.5% in 1,489 randomly selected dairy herds 10() and an apparent prevalence of 37.9% in 290 self-selected dairy herds [16].

*Coxiella burnetii* is enzootic worldwide, yet estimations of prevalence among both human and bovine populations vary widely in the literature. Although *Coxiella burnetii* is a notifiable disease in 29 EU countries, surveillance is passive meaning most data are case based [6].

The EU average for *Coxiella burnetii* antibody prevalence among all cattle is reported to be 15.1% [6]. Ryan et al. [10] had reported a significantly higher risk of *Coxiella burnetii* among dairy cattle than beef cattle which could account, at least in part, for the higher prevalence noted in this study. Pandit et al. [17] reported a prevalence of 69.3% using ELISA BMT testing in Western France dairy cattle. Yanez et al. reported a prevalence of 60.1% using ELISA BMT in dairy cattle in Northern Spain [18].

Increasing herd size was found to be associated with significantly (*p* < 0.0001) higher odds of BMT *Coxiella burnetii* antibody positivity, echoing prior Irish studies [10, 16]. This is particularly pertinent for the Irish context as the Irish dairy cattle industry has undergone a period of significant growth and expansion since the abolition of EU milk quotas in 2015. During this period, the Irish national dairy cattle herd has increased by 25%, demonstrating an increase each year. Furthermore, in the same period the Irish suckler herd (breeding beef cows) population has declined by over 18%. With previous research indicating that dairy cows are significantly more likely to test positive for *Coxiella burnetii*, this highlights the potential increasing prevalence of Q fever among Irish dairy cattle due to demographic shifts.

The EBI is a composite index that places economic value on genetic merit of dairy cattle. In this study, higher herd average EBI was associated with reduced odds of seropositivity. This is an interesting finding and warrants further examination specifically investigating the health sub-index within the overall EBI value.

The percentage of first-lactation cows was negatively associated with *C. burnetii* bulk milk tank seropositivity. In Ireland the majority of dairy farmers breed their own replacement heifers, so in this context proportion of the herd at first lactation is an indicator of age of herd rather than purchase of non-homebred animals [19]. This is in keeping with the literature where older animals are reported to be more likely seropositive due to cumulative exposure over time [1].

Herds that were unvaccinated and tested positive for IBR had significantly lower odds of Q fever positivity compared to vaccinated herds, whereas herds unvaccinated and negative for leptospirosis and salmonella also had lower odds. Given the observational, cross-sectional design, these associations likely reflect differences in herd management—such as greater animal movement for replacement stock or contract rearing—rather than direct vaccine effects.

Many of the large human outbreaks of Q fever have been traced to small ruminant farms such as the Netherlands 2007–2010 outbreak [20]. Irelands dairy industry is dominated by cattle withminimal dairy goat or dairy sheep farms. Genotyping studies suggest potential host-specific *C. burnetii* strains (e.g. ST20 in cattle vs. ST8 in goats) [21], which may confer different zoonotic risk (reference). Establishing a One Health pathogen genotype database would enable rapid and accurate source detection enabling faster implementation of outbreak control measures as well as prevention measures.

The high prevalence of Q fever in Irish dairy cattle herds warrants further investigation and underscores the value of bulk milk tank testing to identify asymptomatic infections [22].

The occupational risk of Q fever is well documented with transmission via birthing fluids, urine, and faeces. Given the high dairy herd prevalence, human Q fever may be underdiagnosed and underreported, particularly among farmers and veterinarians. Farmer education is essential, as many are unaware of zoonotic risks from seemingly healthy animals (reference). Critically, vulnerable groups, like pregnant women or those with vascular conditions, should avoid animals during calving season [23]. Hygiene measures and personal protective equipment during calving are critical.

The large sample population included in this study is a significant strength. Limitations include regional data coverage, potential sampling bias from programme participants and the fact that bulk milk tank testing detected herd exposure.

Future research should include a spatiotemporal analysis of human Q fever notifications in Ireland alongside, bovine prevalence, include small ruminant populations, investigate vaccination patterns and herd management effects and incorporate genotyping of animal and human isolates to clarify transmission pathways and inform One Health strategies.

## Conclusion

In conclusion, this study revealed a high herd-level prevalence of *Coxiella burnetii*, with larger herd size, lower proportion first lactation cows, lower herd average EBI and certain vaccination practices associated with herd level seropositivity. Bulk milk tank testing proved an efficient, cost-effective method. Strengthened collaboration across veterinary and human health sectors, supported by ongoing research, is essential to guide targeted interventions and reduce zoonotic Q fever risk.

## Supplementary Information

Supplementary Material 1.

## Data Availability

The datasets generated and/or analysed during the current study are not publicly available but are available from the corresponding author on reasonable request.

## Abbreviations

BMT: Bulk milk tank
EBI: Economic Breeding Index
ELISA: Enzyme-linked immunosorbent assay
IBR: Infectious bovine rhinotracheitis
IFA: Immunofluorescent assay
