# Supplementary material for: Q fever in the Irish dairy herd
Source: Ir Vet J. 2026 Apr 15;79:28. doi: 10.1186/s13620-026-00343-5 (PMC13196157; doi:10.1186/s13620-026-00343-5)
Supplement: Supplementary file 1 — Supplementary Material 1. [file 13620_2026_343_MOESM1_ESM.docx]

**Supplementary material**


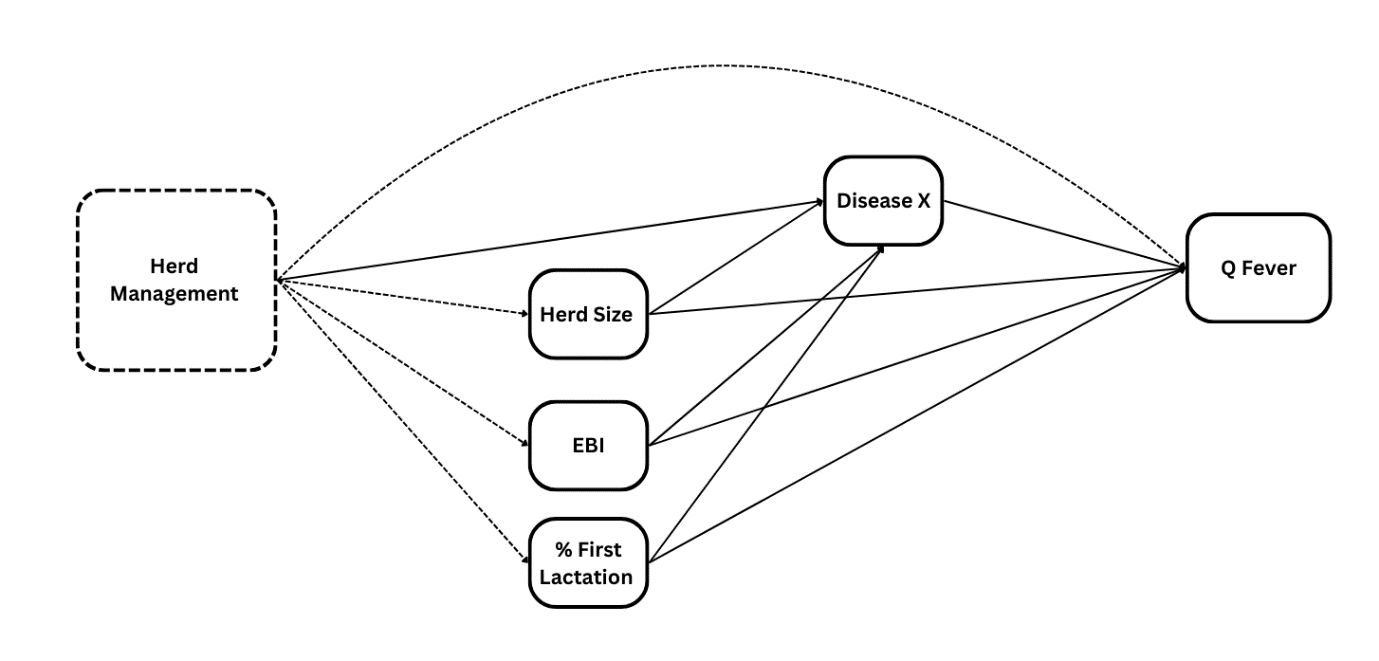


Figure 1. Directed Acyclic Graph (DAG) illustrating potential causal pathways linking herd management, herd-level characteristics, and co-infections to Q fever seropositivity in dairy cattle.

Solid lines represent hypothesized direct causal effects, while dashed lines indicate indirect or potential confounding pathways.
